# COVID-19, Telemedicine and Emergency Department Referrals: Patient Presentations and Follow-up Times to a Community Mental Health Team

**DOI:** 10.1192/j.eurpsy.2022.540

**Published:** 2022-09-01

**Authors:** M. Zubir, J. Costello, A. Ali, C. Erwins, M. Cheasty, L. Judge

**Affiliations:** 1 The Rotunda Hospital, Specialist Perinatal Mental Health Service, Dublin, Ireland; 2 Health Service Executive, North Dublin Mental Health Service, Dublin, Ireland

**Keywords:** Telemedicine, AUDIT, Covid-19, Community Mental Health Services

## Abstract

**Introduction:**

The COVID-19 pandemic caused changes to how healthcare services are utilised and delivered.

**Objectives:**

We examine the impact of COVID-19 on the pattern of emergency patient presentations referred on to the community mental health team and the impact of utilising telemedicine on time to follow-up.

**Methods:**

We retrospectively reviewed all clinical records of patients currently attending our service. We identified presentations to the emergency department (N=119) who were subsequently referred on for mental health follow-up.

**Results:**

Patients being referred to our team from emergency departments were significantly younger during, mean age 33.1 years (SD=12.3) compared to before the pandemic, mean age 40.0 years (SD=14.5), p=0.006 and a higher proportion were new patients during, 55.8%, compared to pre-pandemic period 33.3%, p=0.015. There was also a higher proportion of patients presenting with suicidal ideation and lower proportions of affective, psychosis and suicidal/self-injurious acts during the pandemic period compared to before, p=0.006. The ratio of female to male patients on the other hand were similar during both periods, p=0.853. There appeared to be no difference in median time to follow-up pre and during the pandemic (6.0 vs 5.5 days, p=0.995). Further analysis also found no significant impact on time to follow-up upon implementing telemedicine consultations, with median days to initial follow-up of 6 days pre-pandemic, 4.5 days during pandemic + prior to telemedicine and 6.5 days during pandemic + telemedicine, p=0.602.

**Conclusions:**

This study provides preliminary data on the impact of COVID-19 on mental health emergency presentations and utilization of telemedicine on time to follow-up by CMHTs.

**Disclosure:**

No significant relationships.

